# Exogenous Interferon-α and Interferon-γ Increase Lethality of Murine Inhalational Anthrax

**DOI:** 10.1371/journal.pone.0000736

**Published:** 2007-08-15

**Authors:** Jeffrey A. Gold, Yoshihiko Hoshino, Marcus B. Jones, Satomi Hoshino, Anna Nolan, Michael D. Weiden

**Affiliations:** 1 Division of Pulmonary and Critical Care Medicine, Oregon Health and Sciences University, Portland, Oregon, United States of America; 2 Division of Pulmonary and Critical Care, New York University School of Medicine, New York, New York, United States of America; 3 J. Craig Venter Institute, Rockville, Maryland, United States of America; Instituto Oswaldo Cruz and FIOCRUZ, Brazil

## Abstract

**Background:**

*Bacillus anthracis*, the etiologic agent of inhalational anthrax, is a facultative intracellular pathogen. Despite appropriate antimicrobial therapy, the mortality from inhalational anthrax approaches 45%, underscoring the need for better adjuvant therapies. The variable latency between exposure and development of disease suggests an important role for the host's innate immune response. Type I and Type II Interferons (IFN) are prominent members of the host innate immune response and are required for control of intracellular pathogens. We have previously described a protective role for exogenous Type I and Type II IFNs in attenuating intracellular *B.anthracis* germination and macrophage cell death *in vitro*.

**Methodology and Principal Findings:**

We sought to extend these findings in an *in vivo* model of inhalational anthrax, utilizing the Sterne strain (34F_2_) of *B.anthracis*. Mice devoid of STAT1, a component of IFN-α and IFN-γ signaling, had a trend towards increased mortality, bacterial germination and extrapulmonary spread of *B.anthracis* at 24 hrs. This was associated with impaired IL-6, IL-10 and IL-12 production. However, administration of exogenous IFN-γ, and to a lesser extent IFN-α, at the time of infection, markedly increased lethality. While IFNs were able to reduce the fraction of germinated spores within the lung, they increased both the local and systemic inflammatory response manifest by increases in IL-12 and reductions in IL-10. This was associated with an increase in extrapulmonary dissemination. The mechanism of IFN mediated inflammation appears to be in part due to STAT1 independent signaling.

**Conclusions:**

In conclusion, while endogenous IFNs are essential for control of *B.anthracis* germination and lethality, administration of exogenous IFNs appear to increase the local inflammatory response, thereby increasing mortality.

## Introduction

*Bacillus anthracis* (*B.anthracis*), a gram positive aerobic spore-forming bacilli, is found ubiquitously in animals and soil and, depending on the site of entry, causes a wide array of diseases in humans [1]. Inhalation of *B.anthracis* spores results in mediastinal hemorrhage, pneumonia and sepsis with a high mortality in spite of appropriate anti-microbial therapy [2]. While vaccination of animals and humans in animal husbandry had nearly eliminated this disease in the United States, anthrax has gained renewed attention as a bioterrorism agent. During the attacks of 2001, 11 people contracted inhalation anthrax, 11 contacted cutaneous anthrax, and hundreds of individuals were exposed to potentially dangerous level of spores [2]. In spite of adequate antimicrobial therapy, inhalational anthrax had a 45% mortality, underscoring the need for better adjuvant therapies in case of future outbreaks [2].

Once inhaled, *B.anthracis* spores are ingested by alveolar macrophages. Spores subsequently germinate into the vegetative form, with resultant production of both Lethal Toxin (LeTx) and Edema Toxin. Lethal Toxin, a zinc metallopeptidase, is capable of disrupting numerous intracellular signaling cascades, including cleavage of MAP kinase kinase (MKK) family members [3]. This has numerous effects on the host including suppression of cytokine production and impairment in macrophage phagocytic capability [4]–[6]. However, the long and variable latency between exposure and development of disseminated disease in humans and primates suggests this disruption is incomplete in nature and raises the possibility that modulation of innate immune pathways as an important target for immunomodulatory therapy [7].

Interferons (IFN) play a prominent role in the host innate immune response to intracellular pathogens. There are two broad categories of IFNs, Type I (IFN-α, IFN-β) and Type II (IFN-γ). Type I IFNs, while traditionally thought to be involved in the host response to viral infections are also involved during the initial stages of infection with intracellular pathogens such as *Mycobacterium tuberculosis* [8], [9]. Interferon-γ is the prototypical Type II IFN. Multiple studies show a prominent role for IFN-γ in controlling intracellular infections including *M.tuberculosis* and *L.monocytogenes* [10]–[14]. Both IFN-α and IFN-γ have been used successfully as immunoadjuvants for pulmonary tuberculosis with treated subjects demonstrating earlier resolution of sputum cultures, earlier radiologic improvement and reductions in BALF cytokine levels [15]–[17].

However, little is known about the role of IFN-α and IFN-γ on the control of *B.anthracis in vivo*. *In vitro*, *B.anthracis* is capable of disrupting IFN signaling. Lethal toxin inhibits LPS mediated Interferon Response Factor-3 production, a prerequisite for IFN−α/β production. In addition, *B.anthracis* inhibits IFN−γ mediated nitric oxide (NO) production and IFN−α mediated STAT1 activation [6], [18]. We have recently described a protective effect for exogenous IFNs during infection with *B.anthracis in vitro*. Exogenous IFN-β or IFN-γ inhibited intracellular *B.anthracis* germination and increased macrophage survival for murine and human alveolar macrophages [18]. Finally, a recent study documents an essential role for IFN-γ producing CD4^+^ cells in the development of effective cell mediated immunity after vaccination with inactivated *B.anthracis* spores [19]. Therefore, the purpose of this study was to determine whether exogenous IFN-α or IFN-γ is capable of protecting mice during infection with *B.anthracis in vivo*.

## Results

**Figure 1:**
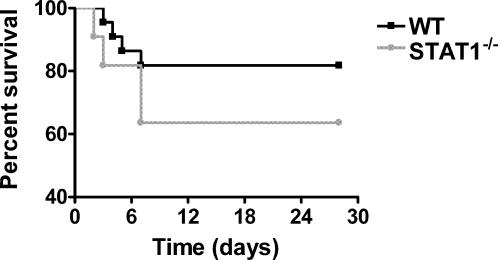
STAT1^−/−^ mice have increased susceptibility to *B.anthracis*. WT and STAT1^−/−^ mice were infected with 10^8^ spores *B.anthracis* intratracheally and monitored for survival. p = 0.13. 8–10 mice/group

**Figure 2:**
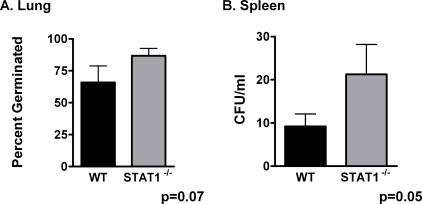
STAT1^−/−^ mice have increased bacterial burden in lung and spleen. Mice were infected with *B.anthracis* (10^8^) and A. Lungs were harvested at 24 hrs and the fraction of germinated spores determined by the formula (CFU *B.anthracis* in lung-CFU *B.anthracis* lung after heat treatment{dormant spores})/CFU *B.anthracis* lung. B. Quantitative culture from spleen harvested at 24 hrs. Serial dilutions were made of whole splenic homogenate. * p<0.05 compared to WT. N = 5/group

**Figure 3:**
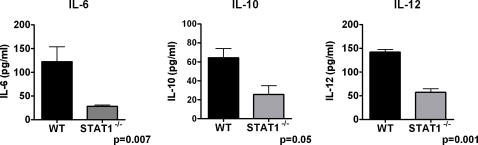
STAT1^−/−^ mice have impaired cytokine induction during infection with *B.anthracis.* Mice were administered *B.anthracis* 10^8^ spores/mouse and plasma harvested at 24 hrs. IL-6/10/12p40 determined by ELISA. N = 5/group.

We first wished to establish an *in vivo* model for pulmonary anthrax. C57BL/6 mice were administered 10^8^ spores 34F_2_ intratracheally. This resulted in significant pulmonary infiltration and PMN recruitment into the alveolar space (data not shown) and an approximate 20% mortality. Saline administration had no effect on mortality or PMN recruitment (Data not shown). Quantitative cultures from whole lung performed 30 min after administration resulted in>90% yield of original inoculums. We next wished to test the role of endogenous IFN in our model. We chose STAT1^−/−^ mice, as STAT1 is required for IFN-α and IFN-γ signaling. STAT1^−/−^ mice had a 2-fold increase in mortality compared to WT mice (37% vs. 19%) with *B.anthracis* infection (Figure 1). This was associated with an increase in the fraction germinated spores within the lung (Figure 2A) as well as extrapulmonary spread as assessed by splenic cultures (Figure 2B). Interestingly, in contrast to WT mice, STAT1^−/−^ mice had marked attenuation in systemic levels of IL-6, IL-10 and IL-12 (Figure 3) suggesting endogenous IFN are required for maximal innate immune activation and cytokine production during inhalational anthrax. Finally, STAT1^−/−^ mice had no difference in total lung Myeloperoxidase activity (MPO) (12.5±30 vs. 8.1±8 pg MPO/Lung; p = NS) compared to WT mice, suggesting little role for STAT1 in controlling PMN recruitment.

**Figure 4:**
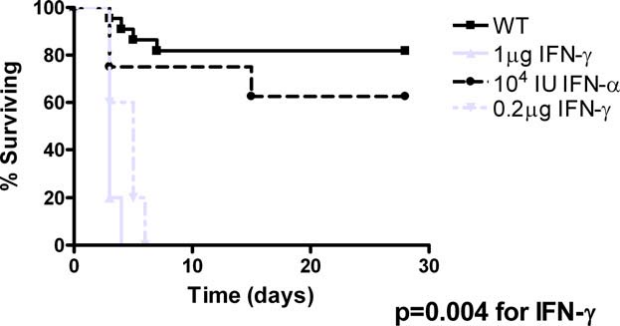
IFN-γ increases mortality in mice with inhalational anthrax. C57BL/6 mice were given 10^8^ spores 34F_2_ intratracheally in 100 µl saline. IFN-γ or IFN-α at described doses was added to saline and administered with *B.anthracis* spores. Mice were subsequently monitored for survival. N = 5–7 mice/group.

Given the requirement of endogenous IFN signaling for optimal control of germination and inflammatory cytokine production *in vivo*, we next wished to assess whether administration of exogenous IFN-α or IFN-γ would improve bacterial control and lethality in our model. Co-administration of IFN-α (10^4^ U) intratracheally with spores slightly increased mortality of *B.anthracis* infection compared to spores alone (38% vs. 18%). However, administration of 1 µg IFN-γ with *B.anthracis* spores resulted in a 100% lethality at 3 days with similar results obtained with 0.2 µg IFN−γ (Figure 4). These effects were specific for the interaction of IFN and *B.anthracis* as mice treated with IFN alone had a 100% survival (data not shown).

**Figure 5:**
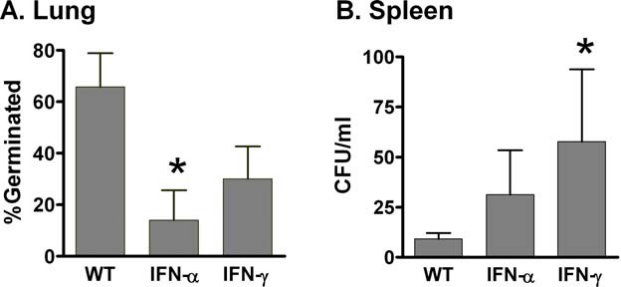
IFN-γ reduces the fraction of germinated spores in the lung. Mice were infected with *B.anthracis* (10^8^)±IFN. A. Lungs were harvested at 24 hrs and the fraction of germinated spores determined by the formula (CFU *B.anthracis* in lung-CFU *B.anthracis* lung after heat treatment{dormant spores})/CFU *B.anthracis* lung. B. Quantitative culture from spleen harvested at 24 hrs. Serial dilutions were made of whole splenic homogenate. * p<0.05 compared to WT. N = 5/group.

We next wished to assess the effect of exogenous IFN on control of *B.anthracis* germination. Neither IFN-α nor IFN-γ had any effect on the total number of viable bacteria and spores recovered from the lung at 24 hrs. However, consistent with previously reported *in vitro* data, both IFN-γ and IFN-α significantly reduced the fraction of germinated spores obtained from the lung (Figure 5A) [18]. In addition, exogenous IFN-γ and to a lesser extent IFN-α, significantly increased extrapulmonary dissemination of *B.anthracis* as determined by quantitative cultures from splenic homogenates (Figure 5B). These changes we not due to alteration in lung PMN content, as WT, IFN-α and IFN-γ treated mice had similar amounts of MPO activity in whole lung (8.1±8.5 vs. 10.2±6.1 vs. 7.2±4.6 pg MPO/Lung; p = NS) and BALF (Not shown).

**Figure 6:**
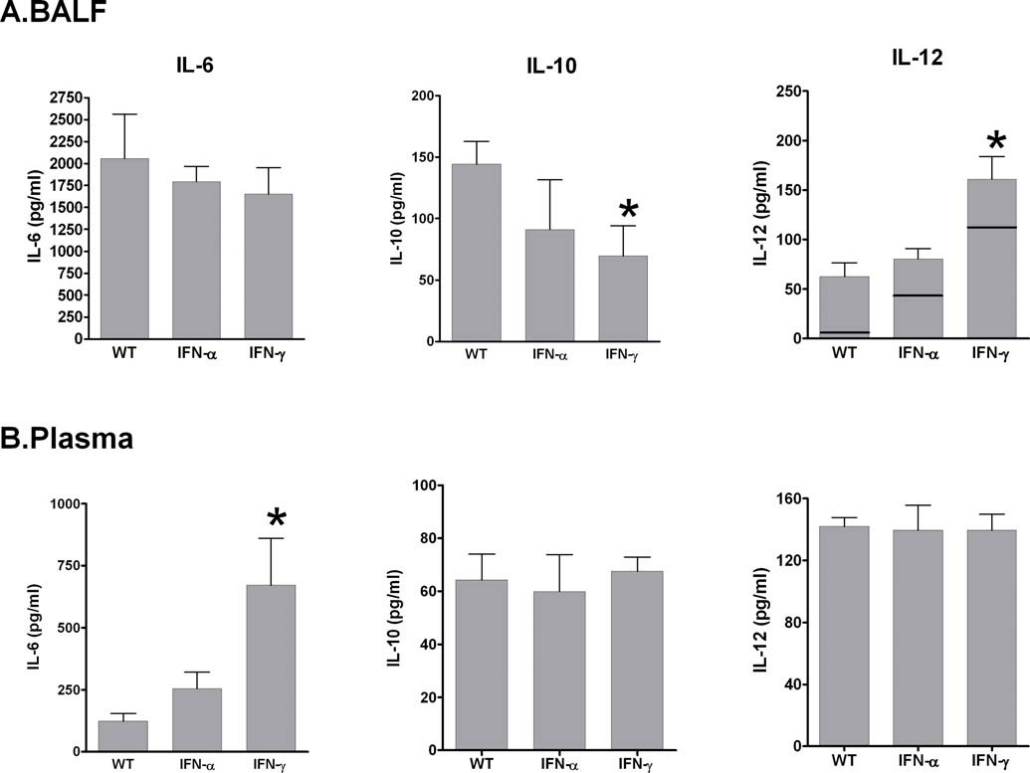
IFN alters the balance of inflammatory cytokines in the lung and serum during infection with *B.anthracis*. Mice were administered *B.anthracis* 10^8^ spores/mouse+IFN-γ (1 µg/ml), IFN-α (10^4^U/ml) or vehicle and BALF and plasma harvested at 24 hrs. IL-6/10/12p40 determined by ELISA. N = 5/group. *p<0.05 compared to WT.- = levels in uninfected mice

The propensity of exogenous IFN to facilitate extrapulmonary spread of *B.anthracis* raised the possibility that IFN stimulation exaggerated the local inflammatory response. Infection with *B.anthracis* resulted in marked upregulation of IL-6, IL-10 and IL-12 in BALF 24 hrs after infection compared to saline treated controls (Figure 6). Exogenous IFNs, in the absence of infection, had no effect on BALF IL-6 or IL-10, but did increase BALF levels of IL-12p40 compared to saline treated controls. This increase in IL-12p40 was further augmented in the setting of inhalation anthrax, especially with IFN-γ. In addition, exogenous IFNs attenuated the *B.anthracis* induction of anti-inflammatory IL-10 in BALF, with no effect on IL-6. In contrast, IFN resulted in marked upregulation of systemic IL-6, with no significant effect on IL-10 and IL-12 (Figure 6). Interestingly, levels of systemic IL-6 correlated with splenic bacterial burden (R^2^ = 0.44; p = 0.02), suggesting IL-6 as a potential marker for extrapulmonary spread of *B.anthracis*.

**Figure 7:**
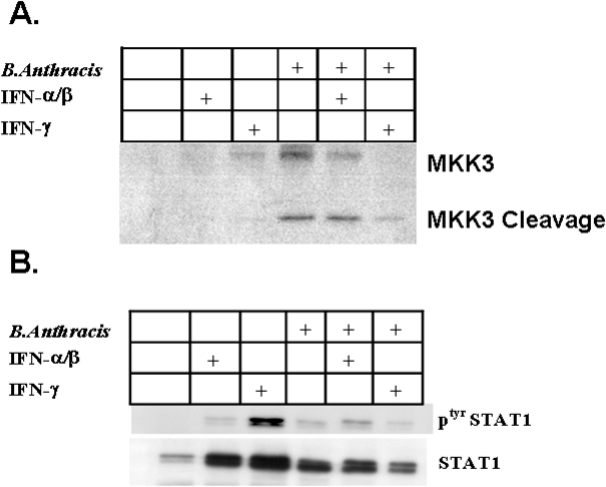
Effect of IFN and *B.athracis* on MKK3 and STAT1 signaling. Mice were administered *B.anthracis* 10^8^ spores/mouse+IFN-γ (1 µg/ml), IFN-α (10^4^U/ml) or vehicle and BALF cells harvested at 24 hrs. For immunoblot. A. Immunoblot for MKK3. B. Immunoblot for phosphor-Tyr^701^ and Total STAT1. Data represent pooled BAL from 5 mice. All lanes were normalized for protein (50 µg/lane)

Finally, we wished to ascertain whether the changes in inflammatory cytokine production were associated with alterations in signaling pathways known to be altered by either *B.anthracis* or exogenous IFN. As expected, infection with *B.anthracis* resulted in cleavage of MKK3 in BALF cells, representing functional LeTx activity *in vivo*. Interestingly, this was attenuated by administration of exogenous IFN-γ (Figure 7A). Similar to our *in vitro* data, infection with *B.anthracis* was only a weak inducer of IFN signaling, as manifest by minimal BALF STAT1 phosphorylation (Figure 7B) [18]. Furthermore, infection with *B.anthracis* inhibited both IFN induced total STAT1 protein expression as well as IFN induced phospho^tyr701^-STAT1 formation, suggesting the phenotypic effects induced by exogenous IFN maybe in part due to STAT1 independent IFN signaling.

## Discussion

One of the major findings of this paper is the sensitizing role for exogenous IFN in the treatment of pulmonary anthrax *in vivo*. Our group has previously described a protective role for exogenous Type I and Type II IFN for macrophages infected with *B.anthracis ex-vivo*, with exogenous IFNs improving both macrophage viability and preventing intracellular germination [18]. Interferons appear to have a similar effect *in vivo*, with IFN treated mice having a significant attenuation in the fraction of germinated bacterium 24 hrs after infection. However, this inhibition was associated with an increase in lethality and extrapulmonary dissemination of *B.anthracis*. The finding of a sensitizing role for exogenous IFN, while surprising, has been described in other infectious models. Exogenous IFN−γ, while improving phagocytosis *in vitro* and *in vivo*, increases mortality in a murine model of invasive aspergillosis [20]. Similar results have been observed in a murine model of invasive candidiasis [21]. In similar fashion, IFN−α mediates the sensitivity to *M.tuberculosis* in mice, with IFN-α/β treated mice having higher bacillary loads and increased mortality [22]. 

There are many potential explanations for this phenomenon. First, is both IFN−α and IFN−γ augment the local inflammatory response within the lung allowing for loss of pulmonary capillary integrity and facilitating extrapulmonary dissemination. The ability of IFNs, especially IFN-γ, to attenuate anti-inflammatory cytokines (IL-10) while maintaining (IL-6) or increasing pro-inflammatory cytokines (IL-12p40) supports this hypothesis. Although we can not exclude this is in part mediated by IL-23, which shares the common IL12p40 subunit, our results are consistent with the observation that exogenous IL-12 administration increases lethality in murine candidiasis by further up regulating endogenous IFN-γ production [23], [24]. The mechanism by which IFN mediates these cytokine effects is less clear. Similar to *in vitro* data, infection with *B.anthracis in vivo* results in only mild induction of IFN signaling as determined by STAT1 phosphorylation. Furthermore, exogenous IFN fails to significantly augment this activation in the setting of infection with *B.anthracis* suggesting a potential for STAT1 independent IFN signaling. While these effects are not due to changes in PMN recruitment, we can not exclude these effects are in part due to differential recruitment of other inflammatory cells to the lung with IFN treatment during infection with *B.anthracis*, including lymphocytes and NK cells. Further studies investigating lineage specific changes in cytokine production and transcriptional activation would help further clarify these changes.

One potential mechanism is restoration of MAPK signaling. Both IFN-α and IFN-γ are known to alternatively signal through MAP kinase family members with resultant inflammatory cytokine production [25]–[27]. In addition, activation of MAP kinase members is required for optimal serine phosphorylation of STAT1 [25], [28]. A hallmark of *B.anthracis* LeTx activity is proteolytic cleavage and inactivation of numerous upstream MKKs, including MKK3 [3]. This results in impaired host response and attenuated cytokine production in response to subsequent inflammatory stimuli [29], [30]. Infection with *B.anthracis* resulted in proteolytic cleavage of MKK3 in BALF. Interestingly, this was inhibited by administration of exogenous IFN-γ suggesting possible restoration of MAPK signaling cascades. Given the observation that MKK3 is required for IL-12 production, this may also explain the preferential effect for exogenous IFN on increasing IL-12p40 levels [31].

However, the sensitizing effect of IFNs in our model does not negate a potentially important role for endogenous IFNs in regulating the host response to *B.anthracis*. The trend towards increased mortality, bacterial germination and dissemination and impaired cytokine production in STAT1^−/−^ mice, suggest an important role for endogenous STAT1 activation in innate immune activation in inhalational anthrax. This is consistent with a recent observation that CD4^+^ cell mediated IFN−γ production is required for optimal cell mediated immunity to *B.anthracis* [19]. However, the mild increase in susceptibility in STAT1^−/−^ mice, compared to the highly lethal phenotype for these mice in *M.tuberculosis*, may provide additional evidence for a role for STAT independent signaling of endogenous IFN in *B.anthracis* [32].

There are limitations to our study. First, these studies used the 34F_2_ strain (Sterne) of *B.anthracis*. Although capable of producing both LeTx and EF, the presence of the former verified by the cleavage of MKK3 in our mice, it is a capsule deficient strain (pXO_2_). This could have significant effects on the ability of IFNs to alter intracellular germination and survival. Therefore, it would be imperative for these results to be repeated with a virulent strain of *B.anthracis* such as Ames. Another limitation is the use of C57BL/6 mice. These mice are known to be more prone to a vigorous Th1 response and are relatively resistant to infection with *B.anthracis* compared to other strains including C3H [33], [34]. Therefore, it is possible exogenous IFNs could have a differential response in other strains. Finally, the use of STAT1^−/−^ mice, while abolishing all typical Type I and Type II IFN signaling, still leaves JAK-STAT independent IFN signaling intact. Further studies, including replicating these experiments in IFN-α/β and IFN-γ receptor deficient mice as well as administration of exogenous IFNs to STAT1^−/−^ mice, will be required to fully ascertain the role of STAT independent IFN signaling in inhalational anthrax.

In conclusion, administration of exogenous IFN-γ, and to a lesser extent IFN-α, while attenuating germination, increases inflammation and extrapulmonary spread of *B.anthracis*. This is in part mediated by STAT1 independent IFN signaling. Further studies are required to determine the role of IFN mediated MAPK activation in *B.anthracis*. Further studies are required, especially with virulent *B.anthracis*, before IFNs can be used as immunoadjuvants for inhalational anthrax.

## Methods

### Mice

6–8 week old Female C57BL/6 mice were obtained from Jackson Labs (Strain #00664). Age and sex matched STAT1^−/−^ mice, bred on C57BL/6 background were obtained from Dr. David Levy at NYU School of Medicine and housed in SPF conditions till use. All experiments were approved by the NYU School of Medicine IUCAC.

### Preparation of *B.anthracis* spores

Sterne strain of *B.anthracis* spores (34F_2_-Coloroda Serum Company) were prepared as previously described [18]. Briefly spores were allowed to germinate overnight at 37°C in Phage Assay broth (8 gm Difco Nutrient broth, 015 gm CaCl_2_, .2 gm MgSO4, 0.05 gm MnSO4, 5 gm NaCl, 10% horse serum). Flasks are then incubated at 30°C for 3–5 days. Spores were spun down and washed with sterile H_2_0×4. Spores are resuspended in sterile H_2_0 and heat treated at 65°C for 30 minutes to kill any vegetative spores. Remaining spores are spun down and washed×2 in sterile water and resuspended in sterile H_2_0 at a concentration of 10^9^/ml. Concentration is determined by quantitative cultures.

### *In vivo* infection model

Mice were anesthetized with 2.5% isoflourane and supplemental oxygen. 100 µl of *B.anthracis* spores or control vehicle were inserted directly into the trachea with a 20 g blunt tip needle. For experiments with exogenous interferon, 10 µl of IFN−γ (provided by Dr. David Levy), IFN−α (R&D systems) or control vehicle was mixed with the spores and inserted simultaneously into the lung. For survival experiments, mice were monitored every 12 hrs for a total of 30 days or until death/sacrifice.

### Quantitative Cultures

Quantitative cultures were made by serial dilutions of whole lung or spleen homogenized in 0.1% Triton X-100. To determine the number of ungerminated bacterium, lung homogenate was incubated at 65C for 30 min, to kill all germinated bacteria.

### ELISAs and Immunoblots

ELISAs for IL-6, IL-10 and IL-12p40 were performed using commercially available ELISAs (R&D systems). BALF cells were lysed in NP-40 lysis buffer as previously described [18]. Immunoblot for total and phosphor-STAT1 and MKK3 were performed on BALF cell lysates as previously described [18]. For all blots, lanes were normalized for total protein. MPO was assessed from whole lung homogenates by commercially available ELISA (Hycult Biotechnologies).

### Statistics

Comparisons between groups was performed with non-parametric T-Test. Survival data was analyzed with Kaplan Meier survival analysis. All data analyzed with Graph Pad 4.0 statistical software.
